# The Skin as an Early Expression of Malignancies in the Neonatal Age: A Review of the Literature and a Case Series

**DOI:** 10.1155/2015/809406

**Published:** 2015-12-21

**Authors:** Vito Mondì, Fiammetta Piersigilli, Guglielmo Salvatori, Cinzia Auriti

**Affiliations:** Department of Medical and Surgical Neonatology, Bambino Gesù Children's Hospital, Piazza S. Onofrio 4, 00165 Rome, Italy

## Abstract

Skin lesions are a frequent finding in childhood, from infancy throughout adolescence. They can arise from many conditions, including infections and inflammation. Most neonatal rashes are benign and self-limiting and require no treatment. Other conditions may be an expression of malignancy or may be a marker for other abnormalities, such as neural tube defects. Therefore, skin lesions require an extensive evaluation and close follow-up to ensure the best possible outcome. This paper briefly reviews the main tumor types presenting with cutaneous involvement in neonates, followed by the description of some patients admitted to our Neonatal Intensive Care Unit with an early skin expression of malignancies.

## 1. Background

Dermatological diseases in neonates are commonly benign and self-limiting, but they may also herald underlying systemic diseases that are potentially life threatening. Neonatal cancer is a relatively uncommon finding; in UK and USA, the incidence is approximately one in every 12,500–27,500 live births [1], varying from 17 to 121 per million live births in reported series [2, 3]. Neonatal cancer, though uncommon, can be already present at birth and a skin lesion can be its first sign. Many malignancies can present already at the first examination after birth as a “blueberry muffin” rash, characterized by localized or generalized reddish-blue papular nodules. In fact, besides congenital infections from the* Toxoplasma*-Rubella-Cytomegalovirus-Herpes (TORCH) complex and severe haemolysis, many early onset malignancies may cause a similar clinical picture (Table 1 and Table 2) [4–9].

In this paper, we review the most frequent neonatal malignancies presenting in the neonate with predominant skin involvement.

## 2. Leukemia

The incidence of neonatal leukaemia, diagnosed in the first 28 days of life after birth, ranges from 1 to 5 million live births [10]. Leukaemia cutis is reported to be the initial presenting sign in 50% of neonates with leukemia [11, 12]. It is a broad term used to describe any cutaneous localisation of leukaemic cells in the skin, occurring in 25–64% of patients with neonatal acute leukaemia. In approximately 10% of the cases, neonates can present with leukaemia cutis, without bone marrow involvement, a condition termed aleukaemic leukaemia cutis [10, 12]. The blueberry muffin rash can be the initial manifestation both of acute myeloid leukaemia (AML) and of acute lymphoblastic leukaemia (ALL). The pathophysiology underlying the migration of leukaemic cells in the skin is not yet understood and there is no evidence of a specific phenotype associated with cutaneous localization. It has been speculated that the chemokine, integrin, and other adhesion (ICAM-1) molecules may play a role in skin specific homing of T and B leukemic cells. CD56 (blast neural cell adhesion molecule) expressed on leukemic blasts has been associated with extramedullary disease in acute leukemia patients with t(8;21) [13]. Nevertheless, some genetic or chromosomal alterations are found in specific subtypes of leukaemia and have a role in conditioning the severity of the clinical course.

Rearrangement of the mixed lineage leukaemia (MLL) gene, which occurs in the 11q23 translocation, leads to aggressive acute leukaemia and may be present in both AML and ALL [11]; it occurs in 34% of this subgroup [14]. On the other hand, spontaneous remission can occur in a subset of neonates affected by AML M4 with t(8;16)(p11;p13). Nevertheless, recurrence can occur in almost half of the cases in the first year of life [15].

In utero leukaemogenesis has been suspected because of the finding of high leukemic cell burdens at birth and from autopsies of stillborn infants with leukemia and because of the description of leukemia with identical karyotype abnormalities in monozygotic twins. An association between maternal exposure to multiple toxins and the development of neonatal leukaemia has been extensively explored and an increased risk related to maternal marijuana use and alcohol consumption has been demonstrated [16, 17]. Therefore, a maternal diet high in flavonoids is suspected to increase the risk of MLL-rearranged infant leukaemia [18].

Congenital leukemia has a higher mortality rate than any other form of congenital cancer; on the other hand, cases of spontaneous remission of AML have been described in patients with AML [19]. These considerations raise the dilemma of deciding when to treat or to wait in case of a neonate presenting with congenital leukemia.

### 2.1. Clinical Presentation

The clinical presentation of neonatal leukemia can be variable. Patients are likely to present with hyperleukocytosis (65% of ALL patients and 49% of AML patients), hepatosplenomegaly (80%), more often than enlarged spleen, central nervous system involvement (50%), and lymphadenopathy (24%). A severe complication of hyperleucocytosis is the leukostasis syndrome, in which white cell plugs are formed in the microvasculature, leading to cardiac failure and respiratory and neurological problems. Neurological symptoms may include somnolence and coma, papilloedema, retinal vein distension, and retinal haemorrhage. Respiratory symptoms may include tachypnoea, dyspnoea, hypoxia with pulmonary infiltrates, or respiratory failure [20, 21]. Furthermore, leukemia may manifest itself with anemia and bone pain because of extramedullary infiltration.

### 2.2. Skin Involvement

**Figure 1:**
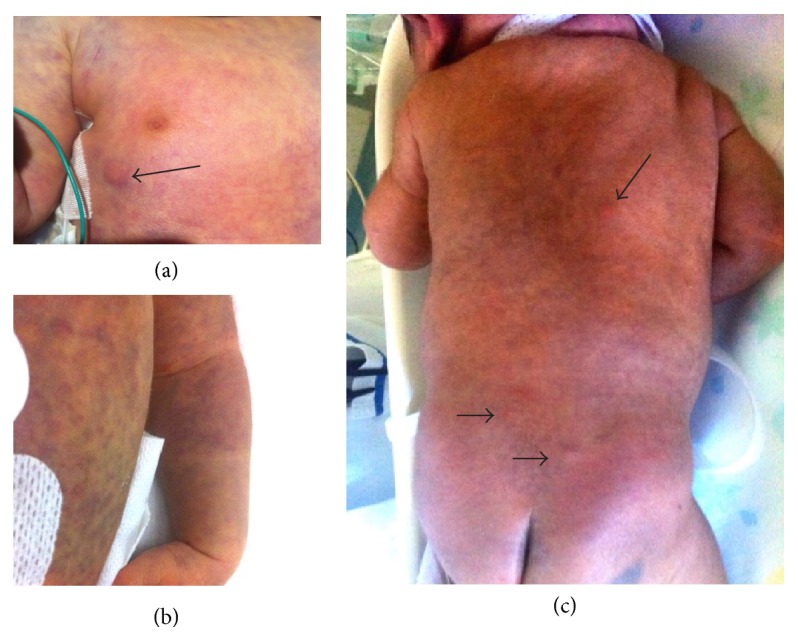
Skin involvement in the leukemia cutis, in a newborn on the first day of life. Randomly distributed subcutaneous nodules (arrows) on the chest (particularly in (a)) and on the trunk (c) with bluish infiltrates on the whole body surface (c) and particularly on the chest and the arm in (a) and (b). The blood cell rose until 167300/mcl, with prevalence of monocytes, low platelets count, and low hemoglobin levels. Infective pathologies have been excluded. The peripheral smear permitted the diagnosis of AML with positivity for CD45, CD33, CD14, CD13 heterogeneous, CD11b, and CD16 dull (AML-4). Genetic analysis revealed t(8;16)(p11;p13). After starting chemotherapy with aracytin, idarubicin, cytarabine, and etoposide, he died at 2 months of life.

Skin involvement is frequently present already at birth with multiple randomly distributed subcutaneous nodules on the trunk and face, with bluish infiltrates on the whole body surface, similar to blueberry muffin eruption, although nodular lesions can be usually small and not bluish, and the infiltrate can be subcutaneous, not nodular and with a generalized distribution (Figure 1). The infiltrate exhibits no blanching response to palpation. In addition to the examination of the peripheral smear to evaluate the percentage of blast cells, thrombocytopenia or anemia, the bone marrow aspirate to perform cytogenetic analysis, a skin biopsy is also mandatory. In approximately 10% of cases of congenital leukemia cutis, cutaneous manifestations may be present in the setting of normal bone marrow and peripheral blood smear [22].

The histopathologic analysis of the lesions in leukemia cutis may reveal several different patterns of infiltration. Most commonly they appear as a dense diffuse dermal infiltrate of pleomorphic leukemic cells, observed in a linear array between collagen bundles in the reticular dermis. Alternatively, band-like as well as compact nodular infiltrates may be seen. These infiltrates rarely reach the epidermis and most cases show extension into the subcutis. The nuclei of these cells may range from markedly atypical folded or reniform nuclei to monomorphous and cytological bands. Determining the leukemic infiltrate subtype almost always requires staining for immunophenotypic markers and their association with cryptologic and cytogenetic data [23, 24].

## 3. Transient Myeloproliferative Disorder

Transient myeloproliferative disorder (TMD) is clonal proliferation of megakaryoblasts, typically occurring in newborns with Down syndrome (DS), with a range between 4% and 6%. The first case was reported by Schunk and Lehman in 1954 [25]. Frequently, TMD disappears during the first 3 months of life, but after a period of normal marrow morphology and peripheral blood count recovery, a significant percentage of patients develop acute leukemia (most commonly AML) within the first 4 years of life. Because TMD appears to be restricted to patients with DS or trisomy 21 (T21) mosaicism, the origin of AML must be related to a cytogenetic abnormality in chromosome 21 [26, 27]. TMD is caused by cooperation between T21 and acquired somatic N-terminal truncating mutations in the key haematopoietic transcription factor GATA1. These mutations, which are not leukaemogenic in the absence of T21, are found in almost one-third of neonates with DS. Analysis of primary human fetal liver haematopoietic cells and of human embryonic stem cells demonstrates that T21 itself substantially alters human fetal haematopoietic development [28].

### 3.1. Clinical Presentation

TMD has variable presentation in the fetus and the newborn from mild disease to disseminated leukaemic infiltration and fulminant hepatic fibrosis. Approximately 10–25% of newborns are asymptomatic and present with circulating blast cells, with or without leucocytosis. Babies with clinical symptoms most commonly present with hepatomegaly, splenomegaly, jaundice, and/or pleural/pericardial effusions. Less common presentations include liver fibrosis, ascites, and renal failure.

The eruption, due to the cutaneous dissemination of the leukemic cells, is apparently more frequent in males and usually develops during the first days of life. Usually it is not associated with fever or respiratory distress, but infants present with high white blood cells count in the peripheral smear. Therefore, in newborns with diagnosed or suspected DS, presenting with cutaneous lesion, TMD needs to be considered as a possible differential diagnosis. The recognition of this rare clinical entity can be important to differentiate other dermatoses requiring totally different management, such as bacterial, viral, or fungal infections, histiocytosis, incontinentia pigmenti, and, of course, leukemia cutis; therefore, further specification of the clinical and histopathological findings is needed.

Because of the possible evolution in acute leukemia DS patients with TMD have to be strictly monitored to eventually start early therapy if needed.

### 3.2. Skin Involvement

**Figure 2:**
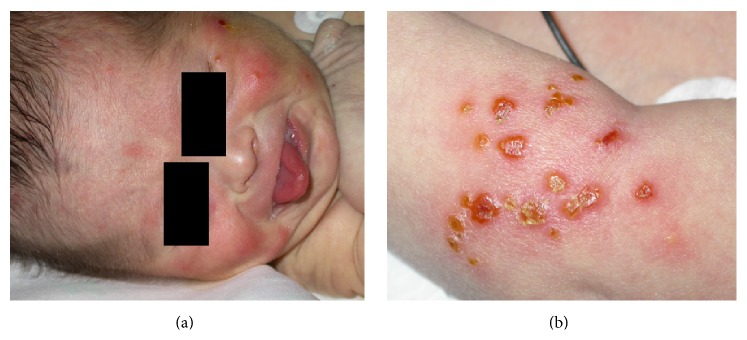
Lesions in a male neonate with Down syndrome, affected by TMD. Gradually, we observed evolution of the skin lesion, such as vesiculopustular eruption on the face (a) and vesiculopustular eruption on arm (b). A biopsy of the lesions showed mild acanthosis epidermis that was focally eroded. Within the superficial dermis and extending into the epidermis were large mononuclear cells with hyperchromatic nuclei and abundant eosinophilic cytoplasm consistent with immature myeloid cells. Immunohistochemical stains showed strong immunoreactivity of these cells with myeloperoxidase and a megakaryocytic cell marker LAT (linker for activation of T cells), confirming that the cells were of myeloid and megakaryoblastic origin (Figure 3). The histologic findings supported the clinical suspicion of a TMD.

**Figure 3:**
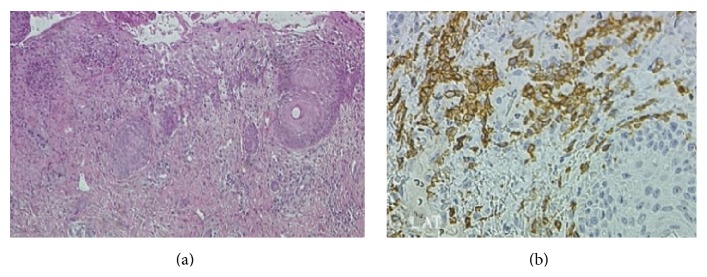
Image of cutaneous lesion biopsy, low-power magnification showing intraepidermal pustule and dermal perivascular infiltrates (hematoxylin-eosin staining) 10x (a). Immunoreaction for LAT (linker for activation of T cells): the cells in the dermis look like atypical blasts of myeloid and megakaryocytic lineage (b).

This unusual form of vesiculopustular eruption is due to the cutaneous dissemination of the leukemic cells and usually develops during the first days of life. The primary lesions are papules, vesicles, or pustules that can rupture and originate erosion and serosanguinous crusts (Figure 2); the face is almost invariably involved, but other lesions often develop elsewhere and can be observed over any skin site, including scalp, palms, and soles. Cutaneous histopathology shows intraepidermal vesicles or pustules (Figure 3) and a dermal mixed inflammatory infiltrate including atypical mononuclear cells. Clearing without scarring is spontaneous over few weeks or months, depending on the course of the underlying haematological disorder, but antiblastic agents and topical corticosteroids have been reported to accelerate the remission of the cutaneous picture.

## 4. Langerhans Cell Histiocytosis

**Figure 4:**
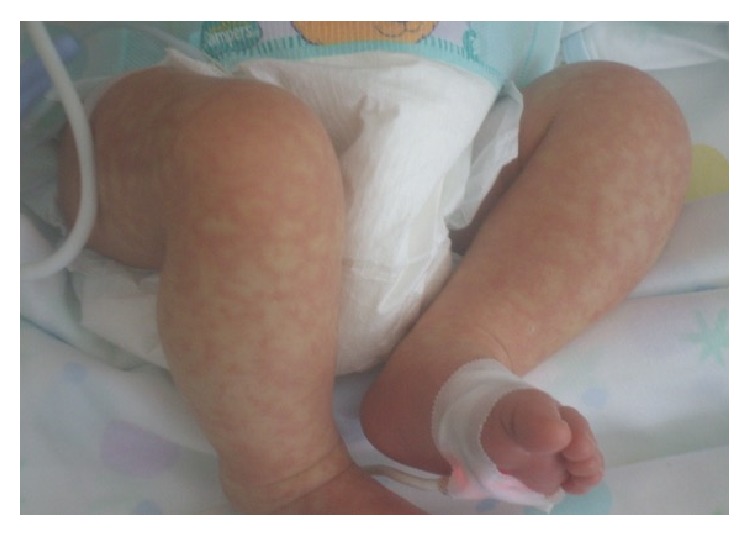
A reticulated, maculopapular rash in the lower limb in a term male affected by haemophagocytic lymphohistiocytosis. He presented with fever associated with generalized rash which disappeared in the first week of life and appeared again on 30th day. Blood test analysis showed values indicative of liver failure; blood cell count revealed low platelets values. The abdominal ultrasound scan showed thinly irregular liver parenchyma. Hypoplasia of granulocyte and erythroblastic and megakaryocytic lineages (<5% each) with haematopoietic cell phagocytosis by mature macrophages were present in the bone marrow aspirate. High-dose glucocorticoids, cyclosporine, and etoposide were started. Despite the therapy, there has been gradual deterioration of the clinical condition until coma, respiratory failure, intractable hypotension, and coagulopathy led to death despite intensive vital support.

The histiocytoses are a diverse group of diseases characterized by the infiltration and accumulation of histiocytes primarily within the blood and tissues. Langerhans cell histiocytosis (LCH) is the main histiocytosis occurring in the neonatal period, followed in order by hemophagocytic lymphohistiocytosis (Figure 4) and juvenile xanthogranuloma [29–32]. Neonatal LCH is rare, with a reported incidence of 1 per 1 million newborns. Approximately 50% of cases involving multiple organs are at higher risk of disease-related mortality [29, 33]. It is a rare disease marked by proliferation of Langerhans-type cells, which shares immunophenotypic and ultrastructural similarities with antigen-presenting Langerhans cells of mucosal sites and skin [34].

The condition was initially described by Lichtenstein in 1953 and referred to as* histiocytosis X* to reflect the uncertain cause and often contrasting features of 3 clinical variants: Letterer-Siwe disease, Hand-Schüller-Christian disease, and eosinophilic granuloma [35]. Recently, the unifying term* Langerhans cell histiocytosis* has replaced* histiocytosis X*, including congenital self-healing reticulohistiocytosis [36], Langerhans cell granulomatosis, and nonlipid reticuloendotheliosis. It is now the preferred terminology of any disorder caused by proliferation of Langerhans cells.

Different oncogenic mutations have been involved in the LCH pathogenesis.

BRAFV600E has been detected in more than half of the cases of LCH in adult age. BRAFV600E mutation results in constitutive activation of the mitogen-activated protein kinase (MAPK) pathway; it has been observed that the MAPK pathway is also activated in cases of LCH without mutations of* BRAF* [37]. Mutations in* MAP2K1*, which encodes the dual-specificity kinase MEK1 protein in the MAPK pathway, have been identified in 27.5% of cases with LCH, thus explaining MAPK pathway activation in the absence of the* BRAF* mutation.* MAP2K1* and* BRAF* mutations were found to be mutually exclusive, as would be expected since MEK1 is directly downstream BRAF within the MAPK pathway [38]. Additional cases of other mutations in the MAPK genes have also been reported, including ARAF and ERBB3 [39, 40].

### 4.1. Clinical Presentation

LCH is characterized by the infiltration and accumulation of histiocytes and other immune effector cells within various tissues. In addition to blue nodules, other typical cutaneous lesions are scaly, erythematous, seborrhoea-like eruptions of brown to red papules. Superficial ulcerations within these lesions are also described resulting in weeping lesions suggestive of eczema.

In addition, patients with multisystem disease have involvement of at least one other system, including the bone, lymph nodes, the central nervous system, the eye, the gastrointestinal tract, the bone marrow, or solid organs. Patients may present with pain, dyspnoea, and failure to thrive [33, 41].

### 4.2. Skin Involvement

Various skin findings are characteristic of LCH. The most common lesions are red brown papules or nodules that may be pseudo vesicular and often crusty. They can be associated with secondary erosion or hemorrhage. In addition, erythematous, scaly dermatitis involving the scalp, posterior auricular regions, perineum, and/or axillae can be seen [42, 43].

A skin biopsy provides a rapid and accessible means to secure the diagnosis. A presumptive diagnosis of LCH may be made based upon light microscopic findings and a compatible clinical picture, but a definitive diagnosis requires that lesional cells exhibit positive staining with S-100 and CD1a, and the identification of Birbeck granules upon electron microscopy is necessary [44].

## 5. Neuroblastoma

Neuroblastoma (NB) is the most common neonatal malignancy. The Automated Childhood Cancer Information System (ACCIS) revealed an incidence of 27% of cancers cases in Europe between 1978 and 1997, with the highest incidence in the first year of life [45]. One study reported that 16% of infant neuroblastomas were diagnosed during the first month of life and 42% during the first 3 months [46]. Stage 4S represents approximately 7–10% of all cases of NB. Most neuroblastomas occur sporadically; familial NB per se is rare and concerns only approximately 1% of all cases [47]. Different genomic mutations have been identified in the last decades. The most important alteration is* MYCN* amplification, which is strongly correlated with advanced disease, drug resistance, and poor outcome [48]. The amplification of* MYCN* and the subsequent overexpression of the protein directly contribute to tumorigenesis. MYCN oncogene is predominantly expressed in the developing peripheral neural crest inducing proliferation and migration, with decreased levels associated with terminal differentiation [49]. Missense mutations of Paired Homeobox 2b (PHOX2B) on chromosome 4p, frequently associated with other neural crest disorders or malignancies (Ondine's and Hirschsprung's disease), were the first germline mutations to be identified in NB predisposition [50–52]. More recently, whole exome and whole genome sequencing analyses have identified loss-of-function mutations/deletions in chromatin modifiers including* ATRX*,* ARID1A*, and* ARID1B*. However,* ATRX* mutations were more common in patients over 5 years of age and no mutations/deletions were identified in the youngest age group (<18 months) [53].

In neonates and infants aged <18 months, NB has a good prognosis as most have favourable biological characteristics and may undergo spontaneous regression even if metastatic. Diagnosis can occasionally be performed during pregnancy by fetal ultrasound [54].

### 5.1. Clinical Presentation

Multiple nodules occur in more than one-third of patients and are characteristic of stage 4S disease. Stage 4S NB is defined by the International Neuroblastoma Staging System (INSS) as metastatic disease with metastases confined to skin, liver, and/or bone marrow in infants younger than one. According to the Stage International Neuroblastoma Risk Group, based on clinical criteria and image-defined risk factors, the age for stage 4S (also called stage MS) has been extended to 18 months [55, 56].

Stage 4S (or MS) can also present with rapid enlarging, diffusively involved liver (“Pepper Syndrome”), which may cause respiratory compromise, renal impairment, and bowel dysfunction together with coagulopathy. NB can also cause spinal cord compression in the neonate, with bladder and bowel dysfunction as well as motor impairment [57, 58].

### 5.2. Skin Involvement

The typical cutaneous eruption is represented by bluish cutaneous nodules with a characteristic blanch response to palpation that leaves surrounding rim of erythema. Ocular signs such as “raccoon eyes,” periorbital ecchymosis, or heterochromia iridis may be present [59].

Histological examination of the cutaneous nodules reveals a uniform, small cell, malignant tumor with or without Homer-Wright pseudo rosette formation. Molecularly, NB stage 4S is characterised by near triploidy and absence of genetic alterations. Structural genetic changes characteristic of stage 4 NB (e.g., segmental chromosomal alterations,* MYCN* amplification, and* ALK* mutations) are not present in children with NB stage 4S, but if they are present, the tumor behaves as if it was a stage 4 NB.

## 6. Rhabdoid Tumor

Rhabdoid tumor (RT) was distinguished from Wilms' tumor in the 1970s. This highly malignant neoplasm is characterized by early metastases and a high mortality rate. The tumor occurs in the perinatal period during the first year of life and occasionally in older children. According to the origin tissue the RT family is divided into 3 categories, with an approximately equal distribution: (1) primary central nervous system lesions, defined as atypical teratoid/rhabdoid tumor, associated with a low incidence of metastases outside the central nervous system, (2) primary renal lesions, and (3) primary soft tissue lesions. The latter typically relates to widespread metastatic disease [60–64]. The incidence of metastatic RT/atypical RT is not defined; however, European data indicate 0.1 to 0.5 per million children per year. Rhabdoid tumors not involving the kidney are very rare [65]. When RT occurs in utero, it is more likely to present at birth with multiple metastases and a rapidly progressive, downhill clinical course ending in early death. Metastatic disease is present in more than half of the neonates at the time of diagnosis.

The vast majority of RTs demonstrate abnormalities in chromosome 22. These abnormalities are characterized by the loss of function of a member of the SWI/SNF chromatin-remodeling complex located at 22q11.2, known with different names (*hSNF5*,* INI1*,* BAF47*, or* SMARCB1*) [66]. In RT, genetic mutations are characterized by somatically acquired biallelic inactivating truncating mutations within the tumor cells, associated or not with a predisposing germline mutation [67, 68].

### 6.1. Clinical Presentation

RT may present in the skin, particularly in the head and neck area, as a solitary primary tumor or as metastatic skin nodules. RTs have to be suspected in case of haematuria with or without hypercalcemia [69]. Neonates can also manifest an increase in cranial circumference due to hydrocephalus, seizures, and irritability because of CNS involvement. Moreover, vomiting, anaemia, fever, and respiratory distress can be present [70].

### 6.2. Skin Involvement

Few case reports describe the cutaneous involvement due to RT [70–72]. Typically primary central nervous system lesions can manifest themselves as one or several lightly erythematous papulonodular lesions. They seem to be verrucous, as observed with verrucous hamartomas.

The histologic diagnosis of RT is based on the identification of the characteristic RT cell, which consists of a round vesicular nucleus, a prominent nucleolus, and round to oval eosinophilic inclusion containing intermediate filaments as seen by electron microscopy [64, 73]. Moreover, the presence of a mutation of the hsNF5/INI1 gene located in chromosome 22q11 is helpful in establishing the diagnosis.

## 7. Rhabdomyosarcoma

Rhabdomyosarcoma (RMS), the most common soft tissue sarcoma, represents 4–8% of all malignant solid tumors in childhood, with few cases described in infants younger than one month of life [74–76]. RMS may be congenital. RMS is traditionally subdivided into embryonal, alveolar, and pleomorphic. The overall prognosis of perinatal RMS is poor, with expected survival around 40% [77].

### 7.1. Clinical Presentation

**Figure 5:**
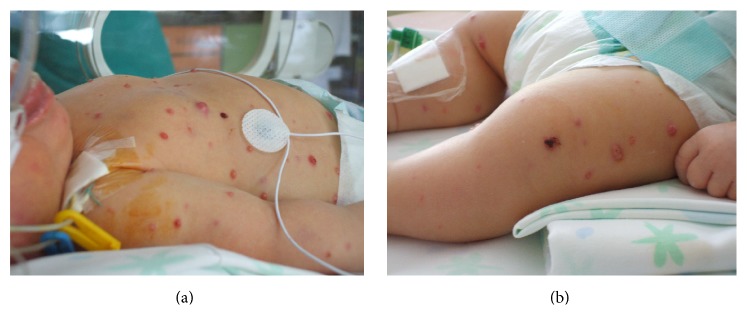
Nodular lesions diffused on the body in a term baby with alveolar rhabdomyosarcoma. Particular image of the chest and right arm (a) and the legs (b). An incisional biopsy of the cutis showed aggregates of atypical small spindle cells interrupted by fibrovascular septae and collagen bands resembling the lung alveoli. Bone marrow biopsy and total body CT scan demonstrated the presence of pulmonary, pleural, and pericardial metastasis. Chemotherapy was started, without a clinical improvement. The baby died at 26 days of life because of renal insufficiency and heart failure.

Most cases of RMS occur in the head and neck region (29.3%) and in the genitourinary tract (26.3%) [78]. Multiple skin nodules can be observed. In addition, neonates can be manifested with haemangioma-like vascular lesions and an abdominal mass; hepatosplenomegaly and thrombocytopenia can be associated. Neonatal Alveolar RMS (Figure 5) frequently manifests itself with multiple skin lesions associated with brain metastasis.

### 7.2. Skin Involvement

RMS is clinically manifested as multiple papulonodular lesions and subcutaneous nodules or as a single papule or nodule, which grows slowly. The lesions may present as soft cherry-red color nodules or as firm violaceous subcutaneous nodules [79].

Most cases of RMS skin metastasis are of the alveolar subtype, with small- to medium-sized darkly stained cells with rounded nuclei and scant cytoplasm arranged in alveolar pattern. It is often very difficult to differentiate the skin metastases of RMS from other skin neoplasms that are composed of spindle or round cells, such as lymphoma, NB, and Ewing's sarcoma [80]. The use of immunohistochemistry may provide additional information to aid in differential diagnosis. Cytogenetic analysis plays an important role in confirming the diagnosis, but only for alveolar RMS because there are no specific cytogenetic or molecular markers for embryonal RMS. Alveolar RMS is associated with specific translocation, t(2;13)(q37;q14) or its variant t(1;13)(p36;q14) [81].

## 8. Other Tumors

Few cases of other neoplasms with cutaneous involvement as first presentation in the first days of life are described. The rare Peripheral Primitive Neuroectodermal Tumor is characterized by the presence of small blue cells in the CNS and in peripheral tissues [80]. Neonatal choriocarcinoma, an aggressive malignant tumor of trophoblastic cells, with cutaneous metastasis has been described in four neonates [82]. The infantile myofibromatosis, the most common fibrous tumor of infancy, is characterized by the presence of myofibroblastic cells that involve the skin, soft tissue, bones, and internal organs [83].

## 9. Conclusion

The blueberry muffin rash as well as nodules, bruising, and crusts may all indicate serious underlying diseases and sometimes may represent the first clinical manifestation of rare neoplasms in the newborn infant. Clinical and laboratory findings can be limited and clinic-pathological correlation is critical. A definitive diagnosis can be obtained only by means of a skin biopsy. Treatment options pose challenges because of the particular vulnerability of the affected neonates. Neonatal cancer raises diagnostic, therapeutic, and ethical issues; management requires a multidisciplinary approach. The prognosis depends on the type of cancer identified, the optimal timing of the therapy, and the subsequent follow-up.

## Figures and Tables

**Table 1:** Causes of the blueberry muffin rash.

Congenital infections	TORCH complex

Severe haemolysis	(i) ABO or Rhesus incompatibility
	(ii) Hereditary spherocytosis
	(iii) Twin-twin transfusion

Congenital vascular lesions	(i) Multiple hemangiomas of infancy
	(ii) Multifocal lymphangioendotheliomatosis
	(iii) Blue rubber bleb nevus syndrome
	(iv) Multiple glomangiomas

Early onset malignancies	(i) Leukemia
	(ii) Langerhans cell histiocytosis
	(iii) Disseminate neuroblastoma
	(iv) Rhabdoid tumor
	(v) Rhabdomyosarcoma
	(vi) Primitive neuroectodermal tumors
	(vii) Choriocarcinoma
	(viii) Myofibromatosis

**Table 2:** Diagnostic tests in malignancies involving the skin, with differential diagnosis (*∗* in addition to the different causes of blueberry muffin baby listed in Table 1; WBC: white blood cell; Hb: Haemoglobin; PLTs: platelets).

Neoplasia	Diagnostic tests	Differential diagnosis^*∗*^
Leukemia	(i) Blood cell count (WBC > 50000; low Hb and PLTs) (ii) Liver function tests (iii) Coagulation studies (iv) Blood film (v) Bone marrow aspirate with morphology, immunophenotype (FAB L1, L2, M4, M5, and M7), and karyotypes [t(4;11)(q21;q23); t(11;19)(q23;p13); t(9;11)(p21;q23); t(1;22)(p13;q13)] of blasts (vi) Skin biopsy	(i) Listeriosis (ii) Sepsis (iii) Intrauterine Parvovirus infection (iv) Congenital HIV (v) Diamond Blackfan anemia (vi) Extramedullary hematopoiesis (vii) Intrauterine or birth-related hypoxia (viii) Neonatal lupus erythematosus

TMD	(i) Blood cell count (WBC 100000–50000 or normal; normal or decreased Hb; and decreased PLTs) (ii) Liver function tests (increased bilirubin and/or transaminases) (iii) Coagulation studies (abnormal coagulation) (iv) Renal function tests (v) Abdominal ultrasound (vi) Echocardiography (vii) Chest X-ray (viii) Blood film (ix) Bone marrow aspirate with morphology, immunophenotype (FAB M7), and karyotypes (GATA 1) of blasts	(i) Nonspecific changes associated with intrauterine growth restriction and trisomies: neutropenia, thrombocytopenia, erythroblastosis, and polycythaemia (ii) Subtle myelodysplastic features: abnormal myeloid cell granulation, giant platelets

LCH	(i) Blood cell count (normal or decreased Hb, RBC, and/or PLTs) (ii) Liver function tests (iii) Coagulation studies (iv) Chest X-ray (v) Abdominal ultrasound (vi) Skin biopsy	(i) Hemophagocytic lymphohistiocytosis (ii) Familial erythrophagocytic lymphohistiocytosis (iii) Infection-associated hemophagocytic syndrome (iv) Hemangioendotheliomas (v) Extramedullary hematopoiesis (vi) Lymphatic malformations (vii) Infantile myofibromatosis (viii) Histiocytomas (ix) Fibrosarcoma (x) Peripheral Primitive Neuroectodermal Tumor

NB	(i) Blood cell count (possible anemia and cytopenias) (ii) Lactate dehydrogenase levels (iii) Ferritin (iv) Liver and renal function tests (v) Urine catecholamines (increased homovanillic and vanillylmandelic acid) (vi) Abdominal and cerebral ultrasound (vii) Chest X-ray (viii) If necessary, brain, neck, and chest MRI (ix) Skin biopsy with karyotypes (MYCN amplification) (x) Bone marrow aspirate	(i) Benign cutaneous epithelioid Schwannoma (ii) Ganglioneuroma (iii) Ganglioneuroblastoma

RT	(i) Urinalysis and renal function tests (ii) Abdominal ultrasound (iii) Chest X-ray (iv) Brain ultrasound (v) If necessary, brain MRI (vi) Skin biopsy with karyotypes (SWI/SNF at 22q11.2)	(i) Wilms' tumor (ii) Medulloblastoma (iii) Sarcomas (iv) Hemangioma

RMS	(i) Blood cell count (normal or decreased PLTs, anemia) (ii) Liver function tests	(i) Wilms' tumor (ii) Sarcomas
